# Is obstructive sleep apnea associated with difficult airway? Evidence from a systematic review and meta-analysis of prospective and retrospective cohort studies

**DOI:** 10.1371/journal.pone.0204904

**Published:** 2018-10-04

**Authors:** Mahesh Nagappa, David T. Wong, Crispiana Cozowicz, Satya Krishna Ramachandran, Stavros G. Memtsoudis, Frances Chung

**Affiliations:** 1 Department of Anesthesia & Perioperative Medicine, University Hospital, Victoria Hospital and St. Joseph Hospital, London Health Sciences Centre and St. Joseph Health Care, Western University, London, ON, Canada; 2 Department of Anesthesia and Pain Medicine, Toronto Western Hospital, University Health Network, University of Toronto, Toronto, ON, Canada; 3 Department of Anesthesiology, Critical Care & Pain Management, Hospital for Special Surgery and Weill Cornell Medical College, New York, NY, United States of America; 4 Department of Anesthesiology, Perioperative Medicine and Intensive Care Medicine, Paracelsus Medical University, Salzburg, Austria; 5 Department of Anesthesia, Critical Care and Pain Medicine, Harvard Medical School, Beth Israel Deaconess Medical Center, Harvard University, Boston, Massachusetts, United States of America; Weill Cornell Medical College in Qatar, QATAR

## Abstract

**Background:**

Difficult airway management and obstructive sleep apnea may contribute to increased risk of perioperative morbidity and mortality. The objective of this systematic review and meta-analysis (SRMA) is to evaluate the evidence of a difficult airway being associated with obstructive sleep apnea (OSA) patients undergoing surgery.

**Methods:**

The standard databases were searched from 1946 to April 2017 to identify the eligible articles. The studies which included adult surgical patients with either suspected or diagnosed obstructive sleep apnea must report at least one difficult airway event [either difficult intubation (DI), difficult mask ventilation (DMV), failed supraglottic airway insertion or difficult surgical airway] in sleep apnea and non-sleep apnea patients were included.

**Results:**

Overall, DI was 3.46-fold higher in the sleep apnea vs non-sleep apnea patients (OSA vs. non-OSA: 13.5% vs 2.5%; OR 3.46; 95% CI: 2.32–5.16, p <0.00001). DMV was 3.39-fold higher in the sleep apnea vs non-sleep apnea patients (OSA vs. non-OSA: 4.4% vs 1.1%; OR 3.39; 95% CI: 2.74–4.18, p <0.00001). Combined DI and DMV was 4.12-fold higher in the OSA vs. non-OSA patients (OSA vs. non-OSA: 1.1% vs 0.3%; OR 4.12; 95% CI: 2.93–5.79, p <0.00001). There was no significant difference in the supraglottic airway failure rates in the sleep apnea vs non-sleep apnea patients (OR: 1.34; 95% CI: 0.70–2.59; p = 0.38). Meta-regression to adjust for various subgroups and baseline confounding factors did not impact the final inference of our results.

**Conclusion:**

This SRMA found that patients with obstructive sleep apnea had a three to four-fold higher risk of difficult intubation or mask ventilation or both, when compared to non-sleep apnea patients.

## Introduction

Obstructive sleep apnea (OSA) is characterized by intermittent episodes of either complete or partial upper airway obstruction resulting in desaturation and recurrent arousal episodes from sleep. In the general population, the prevalence is 9–25%[1] with a higher prevalence in the bariatric surgical population.[2] Despite the strong association between OSA and adverse perioperative complications,[3–5] the majority of OSA cases remain undiagnosed and untreated at the time of surgery,[6] The difficult airway in OSA patients is considered to be a main contributing factor to the higher rate of adverse respiratory events.[7]

Difficult airway can come in the form of either difficult intubation, or mask ventilation, or a combination of both. Although the incidence of a difficult intubation (1–6%) and failed intubation (0.1–0.3%) are very low,[8,9] it can contribute to increased risk of airway trauma, rapid desaturation, laryngeal injuries, unexpected intensive care unit admission and death.[10,11] Difficult airways may be secondary to upper airway abnormalities like short, thick neck, restricted neck extension, decreased jaw movement and poor tissue mobility. The majority of difficult or failed intubations are precursors of life-threatening airway complications.[10]

OSA is considered to be an important risk factor for difficult airway management.[12–27] Studies have shown that OSA patients are at increased risk of either difficult intubation[12,13,15,18–23,25–27] or difficult mask ventilation[16,18,19,22,24,25] or both.[17,22] American Society of Anesthesiologists Task Force recommended that patients with known or suspected OSA may have difficult airways and therefore should be managed according to difficult airway management guidelines.[28,29] The different abnormalities in the upper airway anatomies like a large tongue, overcrowding of the oropharyngeal structures, decreased upper airway diameter and greater neck circumference may contribute to the difficult airway in OSA patients during the perioperative period.[30–32] Lateral cephalometric studies of the upper airway confirmed cranio-cervical and mandibulo-hyoid deformities in both OSA and difficult airway patients.[33–35] These shared upper airway abnormalities may contribute to the increased risk of a difficult airway being encountered in OSA patients and vice versa.[12,23]

The ASA Task Force[28,29] provided consensus of association, but failed to provide the strength of evidence in the form of odds ratio between OSA and difficult airway. A number of other studies investigated the association between OSA and the occurrence of a difficult airway in patients undergoing various types of surgical procedure.[12–27,36] These investigations have shown a consistently positive association between OSA and difficult airway. However, strong meaningful conclusions cannot be drawn as the studies differed in type, prevalence of OSA, methodologies, sample sizes, data limitations, outcome definition, and variations in the magnitude of associations. The objective of this meta-analysis is to determine the evidence between difficult airway and OSA surgical patients compared to non-OSA patients. Therefore, we hypothesized that the presence of OSA is significantly associated with a difficult airway in patients undergoing surgery. This quantitative review is prepared as part of the work of the Society of Anesthesia and Sleep Medicine’s committee on development of a guideline for intraoperative management of adult patients with OSA.

## Methods

### Literature search strategy

A systematic search was performed to identify prospective or retrospective cohort studies related to OSA and difficult airway management. The following databases were systematically searched through from 1946 to April 2017 for relevant studies: EMBASE, Medline (via PubMed), Cochrane Central Register of Controlled Trials, Medline In-process, Cochrane Database of Systematic Reviews, and CINAHL. Continued literature surveillance and update was done up to October 2017.

The search included the combination of the following MESH key words: “sleep apnea, obstructive”, “obstructive sleep apnea”, “obstructive sleep apnea syndrome”, “sleep disordered breathing”, “obesity hypoventilation syndrome”, “apnoea or apnea”, “hypopnoea or hypopnea”, “anesthesia”, “anesthesia, general”, “airway”, “airway extubation”, “airway management”, “Intubation”, “Intratracheal”, “difficult ventilation”, “Laryngeal Masks” and “face masks”. The selection of the studies was not restricted by country of origin. Two authors independently performed the literature search (MN and DW) and the articles obtained from the search were reviewed. To ensure complete search of literature, citation search was performed on the relevant articles. First the abstracts, then the full-text of the selected studies were inspected separately by two reviewers (MN and DW) to decide the inclusion criteria. Any disagreements were resolved with the consultation of another author (FC).

### Study selection criteria

Articles were assessed by two authors (MN and DW) independently. The inclusion criteria are: 1) adult surgical population aged ≥18 years; 2) polysomnography, chart documentation or screening questionnaires available to diagnose or suspect OSA; 3) report on at least one difficult airway event [either difficult intubation (DI), difficult mask ventilation (DMV), failed supraglottic Airway insertion and surgical airway] in OSA compared to non-OSA patients; and 4) either prospective or retrospective cohort studies. Disagreements regarding the inclusion of the articles were resolved by consulting other co-authors.

### Data extraction

Data extracted included: study author, study year, study type, country of origin, suspected or diagnosed OSA (polysomnography, chart or clinical diagnoses and screening questionnaires), difficult airway events (e.g. difficult intubation, difficult mask ventilation, failed supraglottic airway insertion and surgical airway), and patient characteristics including: total sample size, sample size in each group (OSA vs. non-OSA), age, male gender, body-mass index (BMI), neck circumference (NC), AHI and type of operation. To assess the quality of the studies included in our Systematic Review and Meta-analysis (SRMA), we rated each study using the Newcastle–Ottawa scale.[37] The study authors were contacted by email for any missing data. If needed, unadjusted odds ratio was manually calculated for inclusion in the meta-analysis, and if this was not possible, the study was excluded.

### Outcome definition

**Difficult Intubation (DI):** Endotracheal intubation was rated as difficult in the presence of poor visualization of the glottis (Cormack and Lehane grade III or IV) or when an intubation aid (stylet, intubating laryngeal mask airway, fiberoptic bronchoscope) was needed, or when three or more intubation attempts were required.[38]

**Difficult mask ventilation (DMV)** was defined as mask ventilation inadequate to maintain oxygenation, unstable MV, or MV requiring two providers or Impossible Mask Ventilation (IMV) noted by absence of end-tidal carbon dioxide measurement and lack of perceptible chest wall movement during positive pressure ventilation attempts despite airway adjuvants and additional personnel.[39]

**Failed LMA** was defined as any acute airway event occurring between insertion of LMA and completion of surgical procedure that required LMA removal and rescue endotracheal tube placement. This included all clinically significant airway events from inadequate ventilation to severe desaturation, hypercapnia, and airway obstruction associated with a failed LMA for which an acute airway intervention was clinically indicated.[14]

### Quantitative data synthesis

The meta-analysis of risk estimates was conducted for difficult airway events and exposure to OSA compared to non-OSA patients. We applied continuity correction to studies with zero events by the addition of 0.5 to all cells for calculation of the odds ratio. We statistically summarized pooled estimates through random-effects models.

Heterogeneity was tested and quantified with the I^2^ statistics respectively.[40] A random-effects analysis was used to estimate the odds ratio. Meta-regression analysis was carried out for each of the baseline confounding factors (as a continuous variable) and on the various subgroups (as a categorical variable) (prospective vs. retrospective study; PSG vs. STOP-Bang; good vs. poor quality of study; presence vs. absence of airway outcome definitions; presence or absence of data on confounding factors, low vs. high prevalence of OSA and based on the sample size). Publication bias was investigated by using the Begg’s test and Egger’s test.[41] Statistical tests were conducted using Review Manager (RevMan 5.3) and OpenBUGS v3.0.[42] Statistical significance was considered if the P value (two-sided) is < 0.05. The study protocol is provided in the “S1 File”.

## Results

**Fig 1:**
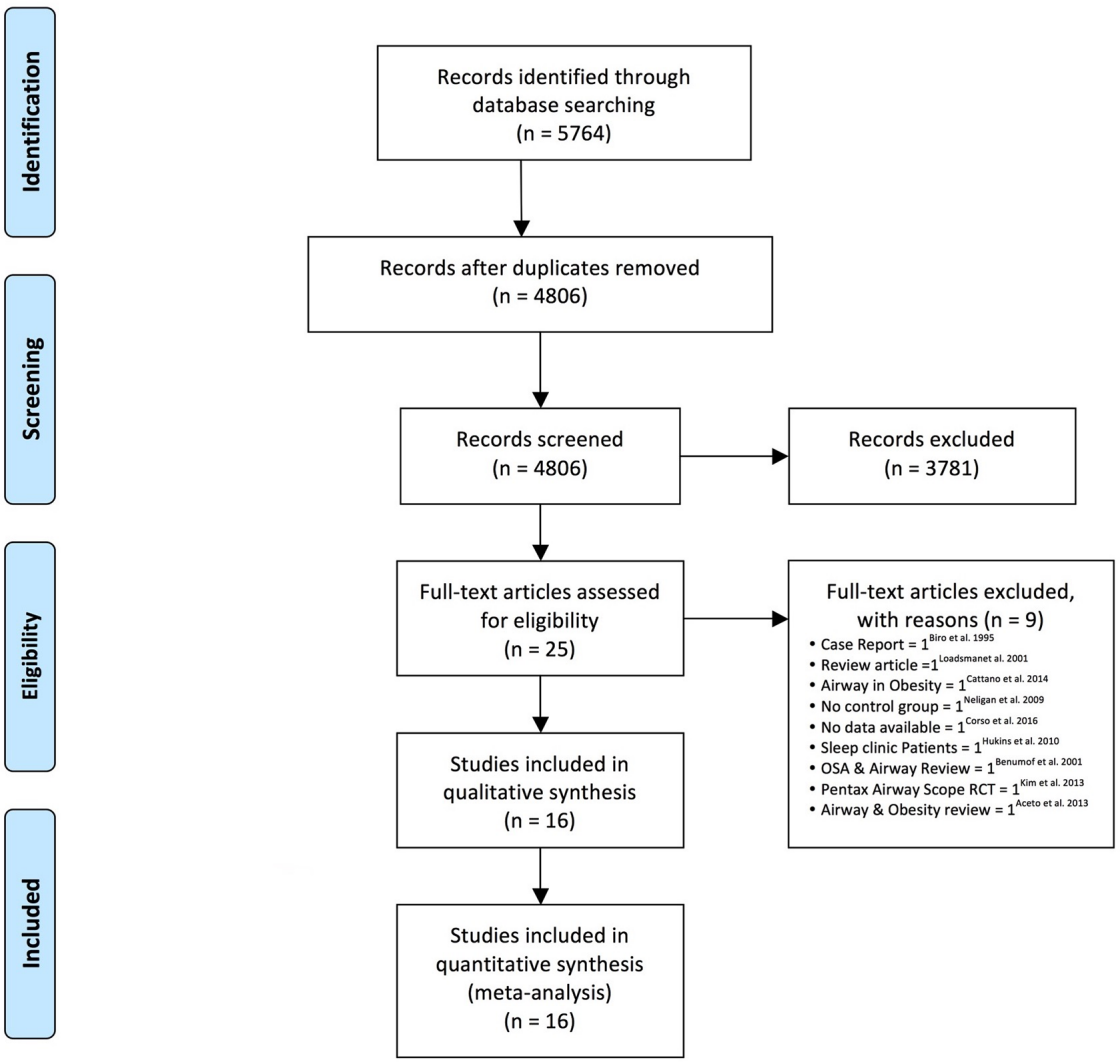
Flow diagram of search strategy used for systematic review and meta-analysis.

Fig 1 summarizes our strategy for literature search. The full search strategy used is shown in the “S2 File”. We identified 4,806 citations and 25 studies were retrieved for complete review and data extraction. Nine studies were excluded (S3 File: Excluded studies). Sixteen studies with a total of 266,603 patients (32,052 OSA vs. 234,551 non-OSA) and a variety of surgical procedures: head and neck, thoracic, abdominal, vascular, genitourinary and orthopedic surgeries were incorporated into the meta-analysis.[12–27] Several studies reported on more than one difficult airway events. Of the sixteen studies, twelve studies provided data on DI (total 19,581: 1,775 OSA vs. 17,806 non-OSA),[12,13,15,18–23,25–27] six on DMV (total 71,489: 5,129 OSA vs. 67,759 non-OSA),[16,18,19,22,24,25] two on combined DI and DMV (total 191,049: 26,361 OSA vs. 164,688 non-OSA),[17,22] and two on failed supraglottic airway (total 15,832: 662 OSA vs. 15,170 non-OSA).[14,21] No study showed data on OSA and surgical airway. Eleven studies were prospective[14,17–20,22–27] and five retrospective in nature.[12,13,15,16,21] The patient characteristics of all included articles are described in Table 1. The summary of clinical characteristics between the OSA and non-OSA group were compared in Table 2.

**Table 1:** Patient baseline characteristics.

Study ID et al.	Location	Diagnosis	Stud Type	Sample size			Age (Mean, years)		Male Gender (%)		BMI (Mean, kg/m^2^)		Neck Circumference (cm)	
				Total (n)	OSA (%)	No OSA (%)	OSA	No OSA	OSA	No OSA	OSA	No OSA	OSA	No OSA
^[^^12^^]^Hiremath^1998^	Australia	PSG	RC	30	33	67	NA	NA	NA	NA	NA	NA	44±2	41±2
^[^^20^^]^Brodsky^2002^	USA	Clinical diagnosis	PC	100	44	56	43±11		22		49±9		45±5	
^[^^13^^]^Siyam^2002^	France	PSG	RC	113	32	68	54±13	53±12	92	88	29±4	25±4	NA	NA
^[^^21^^]^Sabers^2003^	USA	PSG	RC	468	50	50	57±12	56±13	73	73	35±7	33±7	NA	NA
^[^^22^^]^Kheterpal^2006^	USA	PSG*	PC	14370	5	95	NA	NA	NA	NA	NA	NA	NA	NA
^[^^15^^]^Kim^2006^	Korea	PSG	RC	180	50	50	44±9	44±9	95	95	27±3	25±3	NA	NA
^[^^23^^]^Chung^2008^	Canada	PSG	PC	33	66	34	60±10	50±17	81	45	32±7	28±8	42±4	37±4
^[^^24^^]^Kheterpal^2009^	USA	PSG*	PC	53041	7	93	NA	NA	NA	NA	NA	NA	NA	NA
^[^^25^^]^Shah et al.^2012^	India	PSG*	PC	500	1	98	NA	NA	65		NA	NA	NA	NA
^[^^14^^]^Ramachandran^2012^	USA	PSG*	PC	15795	4	96	NA	NA	NA	NA	NA	NA	NA	NA
^[^^17^^]^Kheterpal^2013^	USA	PSG*	PC	176,679	14	85	NA	NA	NA	NA	NA	NA	NA	NA
^[^^27^^]^Acar^2014^	Turkey	SB	PC	200	41	58	55±14	40±13	54	31	30±5	25±4	42±5	38±4
^[^^16^^]^Cattano^2014^	USA	PSG*	RC	1399	17	83	NA	NA	NA	NA	NA	NA	NA	NA
^[^^19^^]^Corso^2014^	Italy	SB	PC	3452	13	87	63±13	58±17	83	48	32±5	25±4	NA	NA
^[^^26^^]^Toshniwal^2014^	USA	SB/PSG	PC	117	80	20	NA	NA	NA	NA	NA	NA	NA	NA
^[^^18^^]^Gokay^2016^	Turkey	SB	PC	126	38	62	60±13	45±12	48	23	29±5	26±4	37± 3	37±3

PSG: Polysomnography; SB: STOP-Bang; PC: Prospective cohort; RC: Retrospective cohort; OSA: Obstructive Sleep Apnea; BMI: Body Mass Index; age, male gender, BMI (≥35kg/m^2^) and neck circumference (cm) are components of OSA.

* Confirmation of Obstructive sleep apnea by electronic data base; Mean ± standard deviation

**Table 2:** Summary of the comparison of baseline clinical characteristics.

^n^Baseline characteristics	OSA	Non-OSA	p value
^**7**^Age (year)^[^^13^^,^^15^^,^^18^^,^^19^^,^^21^^,^^23^^,^^27^^]^	56±6 n = 968	50±7 n = 3604	<0.0001
^**7**^Male gender^[^^13^^,^^15^^,^^18^^,^^19^^,^^21^^,^^23^^,^^27^^]^	78% 756M/212F = 968	51% 1837M/1767F = 3604	<0.0001
^**7**^BMI(kg/m^2^)^[^^13^^,^^15^^,^^18^^,^^19^^,^^21^^,^^23^^,^^23^^]^	31±2 n = 968	27±3 n = 3604	<0.0001
^**4**^Neck Circumference (cm)^[^^12^^,^^18^^,^^23^^,^^23^^]^	41±5 n = 163	38±4 n = 226	<0.0001

n: number of studies which provided the data on the clinical characteristics; OSA: Obstructive Sleep Apnea; BMI: Body Mass Index;* p-value<0.0001; mean ± standard deviation. age, male gender, BMI (≥35kg/m^2^) and neck circumference (cm) are components of OSA.

Pooled analysis showed that the baseline parameters were different between the OSA and non-OSA groups for the following variables: age, male gender, BMI, and neck circumference. A detailed systematic review of the 16 studies is described in the tabular column in “S4 File” and the assessment of the quality of the studies is summarized in the table in “S5 File”. The definition of the airway outcomes used in these studies are provided in the table in “S6 File”.

### Difficult intubation

**Fig 2:**
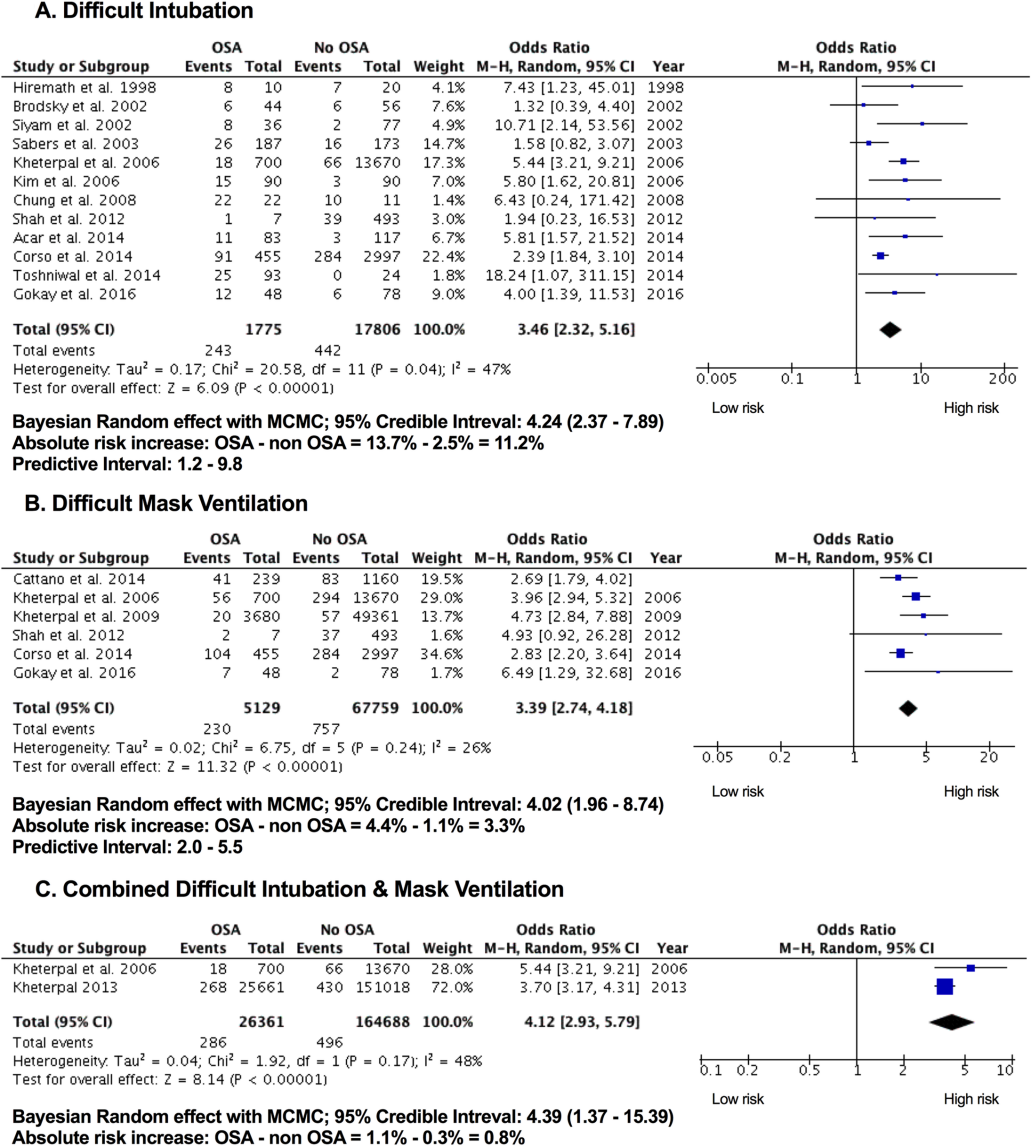
Meta-analysis of difficult airway between OSA and non OSA patients undergoing surgery. The odds ratio of each included study is plotted. A pooled estimate of overall odds ratio (diamonds) and 95% confidence intervals (width of diamonds) summarizes the effect size using the random effects model. CI = confidence interval; M-H: Mantel-Haenszel; OR = Odds ratio; OSA = obstructive sleep apnea.

Fig 2 summarizes the results regarding difficult intubation. Overall in patients with OSA the odds for DI were increased by a 3.46-fold compared to patients without OSA (OSA vs. non-OSA: 13.5% vs. 2.5%; pooled OR 3.46; 95% CI: 2.32 to 5.16, P <0.00001, I^2^ = 47%). The mean estimate varied from study to study, while the lowest was 1.32 for Brodsky et al.[20] and the highest was 18.24 for Toshniwal et al.[26] Although the confidence intervals of the individual studies varied, the mean effect estimates of all the included studies were in the same direction supporting the association of difficult intubation and OSA. Sensitivity analysis revealed two studies as potential sources of heterogeneity[22]^,^[19] and both were also partial outliers in the funnel plot. When the two studies were excluded, the overall effect estimate increased to 3.57 (2.08 to 6.11) and heterogeneity decreased to 32% (results not shown). No evidence for substantial publication bias was found by the Begg’s test (p value = 0.53713) or Egger’s test (0.13745). As per classic fail-safe N test, the number of missing studies that would bring the p-value to more than alpha is 240, confirming the absence of publication bias.

### Difficult mask ventilation

Six studies including 72,888 patients (OSA vs. non-OSA: 5,129 vs. 67,759) provided the data on the DMV (Fig 2).[16,18,19,22,24,25] Overall, DMV was 3.39-fold higher in the OSA than non-OSA patients (OSA vs. non-OSA: 4.48% vs. 1.11%: pooled OR 3.39; 95% CI: 2.74 to 4.18, P <0.00001, I^2^ = 26%). The mean estimate varied from study to study, the lowest was 2.83[19] and the highest was 6.49.[18] Even though the confidence intervals of the individual studies varied, the mean effect estimates of all the included studies were in the same direction suggesting the association of difficult mask ventilation and OSA. Influential analysis revealed one study as a potential source of heterogeneity.[19] When this study was excluded, the overall effect estimate increased to 3.71 (2.98 to 4.62) and heterogeneity decreased to 3%. No evidence for substantial publication bias was found by the Begg’s test (p value = 1.00) or Egger’s test (0.27151). As per classic fail-safe N test, the number of missing studies that would bring the p-value to more than alpha was 199, confirming the absence of publication bias (results not shown).

### Combined difficult intubation and mask ventilation

Combined DI and DMV is 4.12-fold higher in OSA than non-OSA patients. The absolute risk increase for OSA was 0.81% compared to non-OSA. (OSA vs. non OSA: 1.11% vs. 0.3%: pooled OR 4.12; 95% CI: 2.93 to 5.79, P <0.00001, I^2^ = 48%) (Fig 2).[17,22]

### Failed supraglottic airway device

Two studies reported the data on failed supraglottic airway insertion (total: 15,832; OSA vs. non-OSA: 662 vs. 15,170). One prospective controlled study[14] reported on LMA Unique^TM^ and the other retrospective study[21] reported on the use of a laryngeal mask airway. No significant difference in the supraglottic airway failure rates exists between the OSA and non-OSA patients (OSA vs. non-OSA: 1.5% vs. 1.0%; pooled OR: 1.34; 95% CI: 0.70–2.59; p = 0.38). The bayesian pooled estimate with 95% credible interval, absolute risk increase for each airway event and predictive intervals are added to each of the forest plot.

### Meta-regression

To address the issue of the differences in the baseline characteristics, we performed a meta-regression analysis for each of these confounding factors (as a continuous variable), measuring its impact on the outcomes. These confounding baseline characteristics slightly changed the odds ratio but did not significantly affect the overall estimate of the outcome (Figure in “S7 File”). Further, to maximally address these and other limitations of observational studies, we performed the meta-regression analysis (as a categorical variable) and sensitivity analysis on the various subgroups (prospective vs. retrospective studies; PSG vs. STOP-Bang; good vs. poor quality of study; presence vs. absence of airway outcome definition; presence or absence of data on confounding factors, low vs. high prevalence of OSA and based on sample size). These factors slightly changed the odds ratio but did not impact the final inference or results of difficult airway for OSA versus non-OSA groups (Table 3). Finally, the robustness of the pooled estimates was checked by influence analyses. Each study was individually omitted from the data set, followed by recalculation of the pooled estimate of the remaining studies in each case.

**Table 3:** Meta-regression and sensitivity analysis of various subgroups.

Measure or outcome	Study characteristics (No. of studies)	Pooled Estimate	95% CI	I^2^ (%)	Meta-Regression	
					Multi-covariant Coefficient [SE]	p-value
Study type	Prospective (8)^[^^18^^–^^20^^,^^22^^,^^23^^,^ ^25^^–^^27^^]^ Retrospective (4)^[^^12^^,^^13^^,^^15^^,^^21^^]^	3.4 4.3	2.1–5.4 1.4–12.7	45 66	-0.040 [1.01]	0.9681
Quality of study**†**	Good (7)^[^^15^^,^,^19^^–^^22^^,^^25^^,^^26^^]^ Poor–moderate (5)^[^^12^^,^^13^^,^^18^^,^^23^^,^^27^^]^	2.8 5.8	1.7–4.6 2.9–11.2	61 0	-0.097 [2.08]	0.9625
OSA Identification	STOP-Bang(4)^[^^18^^,^^19^^,^^26^^,^^27^^]^ PSG (7)^[^^12^^,^^13^^,^^15^^,^^21^^–^^23^^,^^25^^]^	3.2 4.1	1.8–5.7 2.0–8.1	31 54	-0.304 [0.91]	0.7388
Difficult airway definition	Yes (11)^[^^12^^,^^13^^,^^15^^,^^18^^–^^20^^,^^22^^,^^23^^,^^25^^–^^27^^]^ No (1)^[^^21^^]^	3.9 1.4	2.6–6.0 0.7–3.8	42 -	-0.722 [2.13]	0.7354
Availability of data on >3 confounding factors*	Yes (7)^[^^13^^,^^15^^,^^18^^,^^19^^,^^21^^,^^23^^,^^27^^]^ No (5)^[^^12^^,^^20^^,^^22^^,^^25^^,^^26^^]^	3.0 3.9	1.9–4.9 1.8–8.5	41 37	0.460 [0.86]	0.5947
Sample size <200	Yes (8)^[^^12^^,^^13^^,^^15^^,^^18^^,^^20^^,^^23^^,^^26^^,^^27^^]^ No (4)^[^^19^^,^^21^^,^^22^^,^^25^^]^	4.6 2.6	2.7–7.7 1.5–4.8	0 73	0.827 [2.21]	0.7092
OSA Prevalence <0.5	Yes (8)^[^^12^^,^^13^^,^^18^^–^^20^^,^^22^^,^^25^^,^^27^^]^ No (4)^[^^15^^,^^21^^,^^23^^,^^26^^]^	3.6 3.7	2.3–5.9 1.1–12.3	51 55	0.043 [1.98]	0.9825

**†**Study quality scores were obtained from the Ottawa-Newcastle quality checking. Study was considered good when assigned score was equal or greater than 8 out of 9.

*Confounding factors = age, male gender, BMI and neck circumference. PSG: Polysomnography; CI: Confidence Interval; I^2^: Heterogeneity; SE: Standard Error

### Prevalence of OSA in patients with difficult intubation

Of the 48 patients with difficult tracheal intubation pooled from two studies,[12,23], 30 were later diagnosed with OSA (Prevalence 62%). Among these 48 pooled patients with difficulty associated with tracheal intubation, 39% had mild to moderate and 23% had severe OSA.

The data was not homogeneous enough to evaluate the association between the severity of OSA (based on AHI) and difficult airway. One retrospective study found that AHI was significantly higher in the difficult intubation group than in the control group (28.4±31.7 vs. 5.9±8.9 events/hr; P<0.02).[12] In another retrospective study, OSA patients with difficult intubation had a higher AHI than OSA patients without difficult intubation (67.4±22.5 vs. 49.9±28.0 events/hr).[15] For OSA patients with an AHI ≤40, AHI 40–70 and AHI ≥70 events/hr, the incidence of difficult intubation was 3.3%, 19.3% and 27.6% respectively.[15] We have reported our findings following the Preferred Reporting Items for Systematic Reviews and Meta-Analyses (PRISMA) reporting guidelines (S8 File: PRISMA Checklist).

## Discussion

To date, this is the first systematic review and meta-analysis comparing the difficult airway between the OSA and non-OSA patients undergoing surgery. We found that difficult intubation, mask ventilation and both difficult intubation & mask ventilation was 3.4, 3.4 and 4.1-fold higher in OSA patients compared to non-OSA patients respectively. There was no significant difference in LMA failure rates in OSA vs non-OSA patients. Meta-regression analysis adjusting for baseline confounding factors and subgroup analysis did not substantially change results. No data is available in the literature on the relationship between OSA and surgical airway.

The association of OSA with difficult airway is an important clinical information to perioperative physicians as it can contribute to increased perioperative morbidity and mortality.[3] Despite advancements in airway equipment, perioperative airway complications are still problematic in OSA surgical patients.[3] Many of the adverse respiratory events reported in OSA patients are mild, transient and reversible like oxygen desaturation; however some are catastrophic events.[4,7,43] This can be either death or anoxic brain injury, having direct association with difficult airway, usually in the form of failed reintubation in the postoperative period.[7,44]

### The baseline confounders

The baseline of age, proportion of male gender, BMI and neck circumference was significantly higher in OSA than non-OSA patients. These baseline confounding factors could be the commonly accepted risk factors for the presence of OSA and were expected to be higher in the OSA group.[45] Risk factors (higher BMI & greater neck circumference) in association with other anatomical abnormalities may contribute to the higher prevalence of difficult airway in the OSA vs non- OSA group. These considerations highlight the importance of identifying OSA and its association with difficult airway, as an important step to improving airway management and decreasing airway related complications.

### Patients with difficult intubation may have OSA

The shared upper airway abnormalities in both difficult airway and OSA patients explain the increased prevalence of OSA in patients with difficult intubation. The retrospective study by Hiremath et al. used AHI ≥10 events/hr for the cut-off as the diagnosis criteria for OSA. Fifty-three percent of the patients with difficult intubation had OSA.[12] Similarly, the prospective study by Chung et al. using AHI ≥5 events/hr found 66% of the patients with difficult intubation who were referred for polysomnography had OSA.[23] Among these patients with difficult intubation, 30% had mild, 18% moderate and 18% severe OSA.[23] Because of this strong relationship, the authors recommended that patients with difficult intubation should be screened for OSA and considered for diagnosis with polysomnography.

### Relation between severity of OSA and difficult intubation

A retrospective study was conducted to evaluate the association between the severity of OSA and the occurrence of difficult intubation.[15] For OSA patients with AHI ≤40 events/hr, the incidence of difficult intubation was the same irrespective of AHI. However, for OSA patients with AHI ≥40 events/hr, the incidence of difficult intubation increased significantly as the AHI increased. This study identified AHI as an important predictor of difficult intubation in OSA patients.[15] The greater anterior mandibular depth, smaller mandibular angle and smaller cervical angle in patients with higher AHI may greatly reduce the skeletal confines of the tongue, which may contribute to higher incidence of difficult airway in severe OSA patients.[12,15]

### Anatomical changes in OSA patients

The network of anatomical changes may explain the strong association of difficult airway in OSA patients. Many of these anatomical changes are “hypothesis generating” rather than “hypothesis proving” findings. Skeletal changes contributing to the difficult airway in OSA patients include decreased length of the mandibular ramus, the increased anterior mandibular depth and increased mandibular angle.[12] These skeletal changes greatly reduce the space available for the anterior displacement of the tongue into the submental space,[12] and can also cause difficulty in negotiating airway equipment in the oropharynx leading to difficult direct laryngoscopy.[46,47] The greater cervical angle and greater cranio-cervical angle to some extent compensates for the decreased oropharyngeal space.[48] However, this postural compensation is lost under the influence of sedatives, neuromuscular blockers and other anesthetic agents leading to upper airway collapse. Overall, a large tongue and oropharyngeal disproportion may contribute to difficult airway in OSA patients.[49]

### Heterogeneity of studies

In the sixteen studies, all difficult airway outcomes showed a low to moderate levels of heterogeneity. This extent of heterogeneity was expected as the included studies were clinically and methodologically diverse. We identified studies presenting a potential source of heterogeneity.[19,22] After excluding these studies, recalculated pooled estimates, slightly increased and heterogeneity greatly decreased, supporting the validity to our results. The meta-regression and sensitivity analyses of the various subgroups and Bayesian model failed to show any significant changes in the results, providing more stability to our results.

### Limitations

Some limitations of our systematic review and meta-analysis exist. First, the studies are mostly prospective or retrospective observational cohorts with no randomized controlled trial. This was not surprising as it is difficult to conduct a randomized controlled trial on the incidence of difficult airway management in OSA vs. non-OSA patients. Although it remains an important caveat that our results are based on observational comparative studies, the consistency of results across various subgroups increases confidence that the findings are robust. Moreover, no randomized controlled trial (RCTs) are expected forthcoming in the literature, so the use of meta-regression analysis signifies an excellent method to determine the best possible evidence on this topic. Second, the studies included both diagnosed and suspected OSA patients with the possibility of incorporating false positive or false negative cases in both groups. Third, many of the included studies did not report the data on baseline confounding factors like age, gender, BMI or neck circumference. The other limitations relate to selection bias, observer bias, and variations in outcome definitions and may have introduced possible bias in pooled estimates and their dispersion. Despite these limitations, our meta-analysis offers an up-to-date analysis of the current literature on the bidirectional relationship between difficult airway and OSA in patients undergoing surgery. In the future, it may be useful to evaluate the subset of OSA patients (based on the severity of OSA) that is prone to difficult airway management with video laryngoscopy.

### Conclusion

This systematic review and meta-analysis analysis suggests that patients with OSA had a three to four-fold higher risk of difficult intubation or mask ventilation or both when compared to non-OSA patients. No significant difference in supraglottic airway failure rates between the OSA and non-OSA patients was found.

## Supporting information

S1 File Study protocol. (DOC)

S2 File Literature search strategy. (DOC)

S3 File Excluded studies. (DOC)

S4 File Association of difficult airway in obstructive sleep apnea patients undergoing surgery: A systematic review: A systematic review in tabular column. (DOC)

S5 File Study quality assessment by Newcastle-Ottawa scale and MOOSE guideline. (DOC)

S6 File Definition of airway outcomes. (DOC)

S7 File Metaregression of the effect of baseline confounding factors (age, male gender, BMI & neck circumference) on the log odds ratio for the occurrence of Difficult intubation in OSA versus non OSA patients. Each circle represents a study, telescoped by its weight in the analysis. The relationship was nonsignificant.
(TIF)

S8 File PRISMA checklist. (PDF)

## Abbreviations

OSA: obstructive sleep apnea
SRMA: systematic review and meta-analysis
DI: difficult intubation
DMV: difficult mask ventilation
CI: Confidence Interval
BMI: body-mass index
NC: neck circumference
AHI: Apnea Hypopnea Index
OR: Odds ratio
