# Supplementary material for: Is obstructive sleep apnea associated with difficult airway? Evidence from a systematic review and meta-analysis of prospective and retrospective cohort studies
Source: PLoS One. 2018 Oct 4;13(10):e0204904. doi: 10.1371/journal.pone.0204904 (PMC6171874; doi:10.1371/journal.pone.0204904)
Supplement: S1 File — (DOC) [file pone.0204904.s001.doc]

**Supplementary “S1 File” Protocol** Study protocol for systematic review and meta-analysis to determine the **Association of Obstructive Sleep Apnea and Difficult airway***

Nagappa et al. **“Is Obstructive Sleep Apnea associated with Difficult Airway? Evidence from a Systematic Review and Meta-Analysis of Prospective and Retrospective Cohort Studies.”**

**Objective**

In this systematic review and meta-analysis, we aim to investigate the association of difficult airway among the **OSA and non-OSA patients**.

**Inclusion criteria**

*Study type*

- All observational cohort studies were eligible to enter meta-analysis.
- We will include all studies which were published as original reports and present information on the difficult airway in OSA and non-OSA population.

*Participants*

- Adult patients (>18 years) of all age groups undergoing surgery will be included.

*Definition of exposition*

- All studies which used the polysomnography or screening tool to divide patients into OSA and non- OSA will be included.

*Outcome variable*

- All studies which reported at least one difficult airway event in OSA and non-OSA groups will be included. The difficult airway event can be either difficult mask ventilation, difficult intubation, failed supraglottic airway event or surgical airway.

Outcome measures

- The OR will either be extracted from the published article or calculated by the authors.
- If the OR is not directly reported or cannot be readily extracted from the published data, the reviewers will contact the corresponding authors for additional information (e.g., data provided in 2x2 contingency tables).

*Publication type*

- Full published papers will be eligible.

*Amendment(s): Full published papers excluding case reports, review articles, and editorials will be eligible. No language restrictions were applied.*

**Search Methods**

We will search the following electronic databases:

- MedLine (via PubMed)
- EMBASE
- Scopus
- Web of Science
- Google Scholar

There will be no restriction on the language of publication. In these databases, we will search according to the thesaurus of the NCBI MESH browser the following terms and combinations of keywords in full text:

The following keywords will be employed:

The search included the combination of the following MESH key words: “sleep apnea, obstructive”, “obstructive sleep apnea”, “obstructive sleep apnea syndrome”, “sleep disordered breathing”, “obesity hypoventilation syndrome”, “apnoea or apnea”, “hypopnoea or hypopnea”, “anesthesia”, “anesthesia, general”, “airway”, “airway extubation”, “airway management”, “Intubation”, “Intratracheal”, “difficult ventilation”, “Laryngeal Masks” and “face masks”.

Additionally, bibliographies of identified publications and published reviews will be hand searched for potentially relevant articles. Authors will be contacted if data, methods and/or parameter definitions provided from the respective studies are unclear.

**Reviews**

All references cited in the identified reviews will be manually searched for potentially relevant studies.

**Data collection**

Two reviewers (MN, DW) will independently scrutinize the list of titles, and if available the abstracts, to determine potential usefulness of the article. Final selection will be based on the full text of potentially relevant articles by the two reviewers independently. In cases of disagreement, both authors will review the materials together until a consensus is reached. Study quality will be measured using the Newcastle–Ottawa scale (Wells et al. 2011).

The following study characteristics will be extracted: study ID, publication year, country, study design, cohort size, demographic data (age, gender, BMI, neck circumference), surgical procedures and difficult airway events. From all eligible studies, relevant data will be abstracted in duplicate, using a standardized data extraction sheet. An independent reviewer will confirm all data entries and will check at least twice for completeness and accuracy.

*Amendment(s): A modified version of Newcastle-Ottawa scale was used to rate quality indicators.*

**Meta-analysis & Meta-regression**

*Dichotomous comparisons*

- Data on numbers of OSA and non-OSA subjects with difficult airway and corresponding crude odds ratios and 95% confidence intervals will be calculated.
- Random-effects models to estimate the pooled odds ratios for risk of difficult airway due to OSA will be constructed across all studies. Random effect Bayesian meta-analysis was conducted to verify the results due to sparse data.

*Amendment(s): The value of the I^2^ statistic was used to select the appropriate pooling method: random-effects models for I^2^>=50%.*

*Assessment of heterogeneity*

- Impact of heterogeneity will be assessed by calculating the I^2^ according to Higgins et al. (Higgins JP et al. 2003).

*Amendment(s): Confidence intervals around the I^2^ were also provided.*

*Influence analysis*

- Robustness of the pooled estimates will be checked by influence analyses. Each of the studies will be individually omitted from the data set, followed in each case by recalculation of the pooled estimate of the remaining studies.

*Subgroup/Sensitivity analyses*

- To identify potential sources of heterogeneity and sources of bias, studies will be stratified by study design, quality of the study, study quality scores, OSA identification method, OSA prevalence, sample size, availability of data on confounding factors and measured outcome definitions.
- Further stratifications will be made by adjustment for confounders, mode of diagnosis, and type of study (Retrospective or prospective).

*Amendment(s): Meta-regression was used to evaluate whether effect size estimates were significantly different by specific study characteristics and quality factors. Meta-regression coefficients and p-values were provided.*

**Evaluation of bias and confounding**

*Publication bias*

- Publication bias will be assessed by inspection of the funnel plot and formal testing for

funnel plot asymmetry, using Begg’s test (Sterne JA et al. 2001).

*Amendment(s): Funnel plot in inverse-V shape was presented.*

**Discussion and Evaluating**

- The results will be critically and integratively discussed.

**References**

Higgins JP, Thompson SG, Deeks JJ, Altman DG (2003) Measuring inconsistency in meta- analysis. BMJ 327: 557-560.

Berlin JA, Longnecker MP, Greenland S (1993) Meta-analysis of epidemiologic dose - response data. Epidemiology 4: 218-228.

Sterne JA, Egger M, Smith GD (2001) Systematic review in health care: investigating and dealing with publication and other biases in meta-analysis. BMJ 323: 101-105.

Wells, G. A., Shea, B., O'Connell, D., Peterson, J., Welch, V., et al. The Newcastle-Ottawa Scale

(NOS) for assessing the quality of nonrandomized studies in meta-analysis. 2011.

http://www.ohri.ca/programs/clinical_epidemiology/oxford.asp
