# Supplementary material for: Is obstructive sleep apnea associated with difficult airway? Evidence from a systematic review and meta-analysis of prospective and retrospective cohort studies
Source: PLoS One. 2018 Oct 4;13(10):e0204904. doi: 10.1371/journal.pone.0204904 (PMC6171874; doi:10.1371/journal.pone.0204904)
Supplement: S2 File — (DOC) [file pone.0204904.s002.doc]

# Supplementary “S2 File”: Search history record for Systematic Review: Obstructive Sleep Apnea + Anesthesia + Airway

(drawn from: <http://www.york.ac.uk/inst/crd/revs11.htm>)

| Review/Search Topic: OSA + Anesthesia + Airway; limited to human, adults, English language, where possible | Searcher: Marina Englesakis |
| --- | --- |
| Investigator(s): Dr Frances Chung | Date: September , 2016 |

| Databases | **Database Dates covered** | **Date Database  was searched** | **# Citations** | **# Duplicate Citations** | **Total Citations remaining** | **Notes/Comments** |
| --- | --- | --- | --- | --- | --- | --- |
| Medline (OvidSP) | 1946 - 2016, Sept. Week #1 | Thursday, September 15, 2016 | 1054 | 0 | 1054 |  |
| ePub Ahead of Print / Medline In-Process & Other Non-Indexed Citations (OvidSP) | 2016 Sept. 14 | Thursday, September 15, 2016 | 201 | -16 | 185 | No limits available |
| Embase (OvidSP) | 1947 – 2016 Sept. 14 | Thursday, September 15, 2016 | 4240 | -817 | 3423 | Embase records only; includes 1092 conference abstracts |
| Cochrane Central Register of Controlled Trials (OvidSP)  CCTR | August 2016 | Thursday, September 15, 2016 | 78 | -12 | 66 | Medline, Embase records removed at source. |
| Cochrane Database of Systematic Reviews (OvidSP)  CDSR | 2005 – 2016 September 8 | Thursday, September 15, 2016 | 48 | -1 | 47 | No limits available; only Full SRs  included |
| PubMed-NOT-Medline (NLM) | 1809 – Sept. 15 2016 | Thursday, September 15, 2016 | 132 | -112 | 20 | No limits available |
| ClinicalTrials.Gov (NIH) | n/a | Thursday, September 15, 2016 | 11 | 0 | 11 |  |
|  |  | **Totals:** | **5764** | **-958** | **4806** | **Results provided in a compressed EndNote Library** |
