# Supplementary material for: Is obstructive sleep apnea associated with difficult airway? Evidence from a systematic review and meta-analysis of prospective and retrospective cohort studies
Source: PLoS One. 2018 Oct 4;13(10):e0204904. doi: 10.1371/journal.pone.0204904 (PMC6171874; doi:10.1371/journal.pone.0204904)
Supplement: S3 File — (DOC) [file pone.0204904.s003.doc]

**Supplementary “S3 File”: Excluded Studies**

The following are the excluded studies.

1. Aceto P, Perilli V, Modesti C, Ciocchetti P, Vitale F, Sollazzi L. Airway management in obese patients. Surg Obes Relat Dis 2013;9:809–15.

2. Benumof JL. Obstructive sleep apnea in the adult obese patient: Implications for airway management. Anesthesiol Clin North America 2002;20:789–811.

3. Biro P, Bloch KE. Case Reports Patient with Obstructive Sleep and Difficult Airway Access. 1995;180:417–21.

4. Corso RM, Cattano D, Buccioli M, Carretta E, Maitan S. Post analysis simulated correlation of the El-Ganzouri airway difficulty score with difficult airway. Brazilian J Anesthesiol 2016;66:298–303.

5. Hukins C. Mallampati class is not useful in the clinical assessment of sleep clinic patients. J Clin sleep Med 2010;6:545–9.

6. Kim MK, Park SW, Lee JW. Randomized comparison of the pentax airway scope and macintosh laryngoscope for tracheal intubation in patients with obstructive sleep apnoea. Br J Anaesth 2013;111:662–6.

7. Neligan PJ, Porter S, Max B, Malhotra G, Greenblatt EP, Ochroch EA. Obstructive sleep apnea is not a risk factor for difficult intubation in morbidly obese patients. Anesth Analg 2009;109:1182–6.

8. Cattano D, Katsiampoura A, Corso RM, Killoran P V., Cai C, Hagberg CA. Predictive factors for difficult mask ventilation in the obese surgical population. F1000Research 2014:1–11.

9. Loadsman JA, Hillman DR. Anaesthesia and sleep apnoea. Br J Anaesth 2001;86:254–66.
