# Supplementary material for: Is obstructive sleep apnea associated with difficult airway? Evidence from a systematic review and meta-analysis of prospective and retrospective cohort studies
Source: PLoS One. 2018 Oct 4;13(10):e0204904. doi: 10.1371/journal.pone.0204904 (PMC6171874; doi:10.1371/journal.pone.0204904)
Supplement: S4 File — (DOC) [file pone.0204904.s004.doc]

**Supplementary “S4 File”:** Association of difficult Airway in Obstructive Sleep Apnea Patients Undergoing Surgery: A Systematic Review: A Systematic Review in tabular column

| **Study ID et al.**  **[Country]**  **{diagnosis of OSA}** | **Sample size**  **[OSA Prevalence]** | **Study purpose** | **Study quality assessment* [Quality score†]** | **Surgical procedures** | **confounders identified** | **Results** |
| --- | --- | --- | --- | --- | --- | --- |
| **Prospective Studies** | | | | | | |
| ^[20]^Brodsky^2002^  [USA]  {Clinical diagnosis} | 100  [44] | Morbid obesity & Tracheal intubation | Y/N/Y [8] | NA | Male gender, OSA, MP, BMI & NC | Neck circumference & Difficult intubation: OR 1.13  **Large Neck Circumference**  •Male gender (p <0.001)  •OSA (p = 0.0372)  •Higher MP (p = 0.0029)  •Grade 3 laryngoscope view (p = 0.0375)  Obesity alone is not a predictor of Difficult airway |
| ^[22]^Kheterpal^2006^  [USA]  {PSG}* | 61,252  [5] | Incidence & Predictors of Difficult & impossible mask ventilation | Y/N/Y [8] | NA | BMI, Age, beard, MP, snoring, OSA | **Total anesthetics: 61,252**  **Total cases of MV: 22,660**  •Grade 4 MV (impossible to ventilate): 37 (0.16%)  •Grade 3 MV (difficult to ventilate): 313 (1.4%)  •Incidence of grade 3 MV: 1.4%  •Difficult MV and DI: 84 (0.37%)  **Independent predictors**  **DMV:** BMI, OSA, MP, NC, beard ^modifiable^, limited jaw protrusion & (snoring, & TM<6cm) ^grade 4MV^  **DI + DMV:** limited mandibular protrusion, OSA, snoring & BMI |
| ^[23]^Chung^2008^  [Canada]  {PSG} | 33  [66] | Difficult intubation may need PSG referral | Y/N/Y [6] | NA | Age, gender, BMI, NC | **OSA (22) vs. No OSA (11)**  Total OSA: 22  •Mild OSA: 10 (64%)  •Moderate OSA: 6 (18%)  •Severe OSA: 6 (18%) |
| ^[24]^Kheterpal^2009^  [USA]  {PSG}* | 53,041  [9] | Predictors & outcomes of impossible mask ventilation | Y/Y/Y [9] | NA | Age, male gender, Neck extension, BMI, OSA etc | Impossible mask ventilation: 77 (0.15%) {1/690 cases}  Impossible MV and DI: 19 {1/2,800 cases}  **Independent predictors**  •Neck radiation changes (p = 0.01)  •Male gender (p <0.001)  •OSA (p <0.001)  •MP III or IV (p <0.001)  •Presence of beard (p <0.001)  •ROAUC: 0.8±0.03 |
| ^[25]^Shah^2012^  [India]  {PSG}* | 500  [1.4] | Incidence & predictors of Difficult mask ventilation & intubation | Y/N/Y [8] | Orthopedics, urological, abdominal, gynecological, cardiovascular & neurosurgical | Snoring, retrognathia, micrognathia, macroglossia, NC, MP, Slux, BMI, neck extension | Difficult MV: 39 (7.8%)  DI: 40 (8%)  Difficult MV + DI: 7 (1.4%)  Impossible to MV and intubate: 1 (0.2%)  **Presence of >2 risk factors (DI)**  •Sensitivity 43%  •Specificity 99%  •PPV 74% |
| ^[14]^Ramachandran^2012^  [USA]  {PSG}* | 15,795  [4] | Predictors & clinical outcomes of failed LMA | Y/N/Y [8] | NA | Age, BMI, male gender, thick neck, OSA | Failed LMA: 170 (1.1%)  Difficult mask ventilation: 95 (5.6% of LMA failure)  **Outcome**  •Hypoxia, hypercapnia, or airway obstruction: >60%  •Inadequate ventilation related to leak: >42%  **4 independent risk factors**  •Surgical table rotation  •Male gender  •Poor dentition  •Increased BMI |
| ^[17]^Kheterpal^2013^ | 176,679  [14.5] | Predictors & outcomes of combined DMV & DI | Y/Y/Y [9] | Elective surgery | Age, BMI  gender, OSA radiation exposure,  MP score,  Neck extension | Combined DMV & DI  Incidence: 0.4%  **Independent predictors**  •Age ≥46year: 1.93 (1.35 – 2.76)  •BMI ≥30: 2.16 (1.58 – 2.94)  •Male sex: 2.46 (1.8 – 3.36)  •Mallampati III or IV: 3.21 (2.45 – 4.22)  •Neck mass or radiation: 2.57 (1.18 – 5.60)  •Thyromental distance: 2.4 (1.68 – 3.44)  •OSA: 1.59 (1.12 – 2.27)  •Presence of beard: 1.64 (1.21 – 2.24)  •Neck extension: 1.47 (1.05 – 2.05)  •Jaw protrusion: 1.47 (1.05 – 2.05) |
| ^[27]^Acar^2014^  [Turkey] | 200  [41.5] | SB and Difficult Intubation | Y/N/N [7] | General, Urological Orthopedics Plastic | Age, male gender, BMI, NC | STOP-Bang ≥3 (83) vs. 0-2 (117)  Difficult Intubation: 13.3% vs. 2.6%, P=0.004  Postoperative Cx: 4.8% vs. 1.7%, P=0.235  OR 2.9; 95% CI 0.52-16.2 |
| ^[19]^Corso^2014^  [Italy] | 3452  [13] | SB and postoperative Cx | Y/Y/N [8] | Abdominal, Head and neck Thoracic Genitourinary Vascular Orthopedics | NA [Age, gender, BMI, OSA risk, ASA class] | STOP-Bang ≥5 (455) vs. 0-4 (2997)  Postoperative Cx: OR 3.98; 95% CI 1.69-9.37  •Respiratory Cx: 23.2% vs. 5.6%, P<0.0001  •Cardiac Cx:19.2% vs. 4.3%, P<0.0001  •Neurology Cx: 1.6% vs. 0.6%, P=0.2263  Difficult intubation: OR 1.86; 95% CI 1.37-2.51  Difficult mask ventilation: OR 2.06; 95% CI 1.51-2.83 |
| ^[26]^Toshniwal^2014^ | 127  [80] | STOP-Bang & predictors of Difficult airway | Y/Y/N [8] | Elective bariatric surgery | Age, Male gender, BMI, NC | OSA (42) vs. HR-OSA (51) vs. LR-OSA (24)  DMVBMR: 16 (38.1%) vs. 22 (43.1%) vs. 3 (12.5%)  DMVAMR: 10 (23.8% vs. 15 (29.4%) vs. 2 (8.3%)  Poor VC exposure: 17 (40.5%) vs. 9 (17.6%) vs. 1 (4.2%)  Difficult BI: 6 (14.3%) vs. 9 (17.6%) vs. 0 (0%)  DI: 11 (26.2%) vs. 14 (27.5%) vs. 0 (0%) |
| ^[18]^Gokay^2016^  [Turkey] | 126  [38] | SB vs. Berlin to predict the respiratory Cx | Y/Y/N [8] | Laparoscopic Cholecystectomy | Hypertension DM CAD Arrhythmias Hypothyroid Smoking [age, sex,,BMI, MS] | STOP-Bang ≥3 (48) vs. 0-2 (78)  Respiratory Cx during Intubation: 18% vs. 3%, P=0.008  Respiratory Cx during extubation: 31% vs. 12%, P=0.012  Respiratory Cx in PACU:  •Apnea: 2.1% vs. 0%, P=0.381  •Bradypnea: 8.3% vs. 0%, P=0.019  •Desaturation: 31.3% vs. 3.8%, P<0.001  •Laryngospasm: 4.2% vs 0%, P=0.143 |
| **Retrospective Studies** | | | | | | |
| ^[12]^Hiremath^1998^  [Australia]  {PSG} | 30  [33] | OSA and Difficult tracheal intubation | Y/N/Y [7] | NA | MP score,  NC,  Head extension, cephalometric data | Difficult intubation (15) vs. Control (15)  AHI: 28.4±31.7 vs. 5.9±8.9 (p<0.02)  OSA: 8 vs 2  Severe OSA (AHI≥40): 5 vs. 0 (p<0.03) |
| ^[13]^Siyam^2002^  [France]  {PSG} | 113  [32] | OSA and Difficult tracheal intubation | Y/N/Y [7] | ENT and non-ENT surgeries | BMI | OSA (36) vs. Control (77)  Difficult intubation anticipated: 53% vs. 5 (p<0.0001)  Difficult intubation: 21.9% vs 2.6% (p=0.003)  Mean AHI = 41.7±22.1  LSAT: 65.9%±18.1 |
| ^[21]^Sabers^2003^  [USA]  {PSG}^b^ | 468  [50] | OSA and unplanned hospital admission | Y/Y/Y [8] | Meniscectomy, TURP, DC, Tooth extraction, Umbilical hernia repair | Age  BMI | OSA (234) vs. Control (234)  •Difficult intubation: 8% vs. 6.4% (NS)  •Fiberoptic intubation: 4.3% vs. 2.3% (NS)  •Failed LMA: 0.5% vs. 0.6% (NS)  •Failed intubation: 1.6% vs. 0.6% (NS) |
| ^[15]^Kim^2006^  [Korea]  {PSG} | 180  [50] | OSA and Difficult intubation | Y/Y/Y [9] | Uvulo  Palato  Pharyngo  plasty | Severity of OSA | OSA (90) vs. Control (90)  Difficult intubation: 16.6% vs. 3.3%  Incidence of Difficult intubation  •AHI >70; (n=29, mean AHI 84±10.9): 27.6%  •AHI 40-70; (n=31, mean AHI 53±7.9): 19.3%  •AHI <40; (n=30, mean AHI 20.8±10.2): 3.3% |
| ^[16]^Cattano et al.^2014^  [USA]  {PSG}* | 1399  [17] | Predictors of Difficult mask ventilation | Y/Y/Y [9] | General surgical population | Age, BMI  NC, OSA, short neck facial hair | **Difficult mask ventilation**  Incidence: 8.9%  **Seven risk factors**  • Age ≥47: 1.97(1.32 – 2.94)  • BMI ≥35: 2.09(1.35 – 3.23)  • Neck circumference ≥40: 2.54(1.59 – 4.05)  • History of Difficult intubation: 4.65(1.2 – 18.02)  • Facial hairs: 2.34(1.43 – 3.83)  • Short neck: 1.88(1.06 – 3.32)  • OSA: 1.65(1.07 – 2.56) |

* Study quality assessment were categorized into 3 components such as (1) Study population clearly identified; (2) Clear definition of outcomes and outcome assessment; (3) Selective loss of patients during the follow up (if one of these key points was not clearly mentioned in a study, it was assigned a “No”). Y: Yes; N: No; † Quality scores were obtained based on Newcastle-Ottawa scale. †† Important confounders and/or prognostic factors identified; NS: Not Significant; SB: STOP-Bang questionnaire; MS Mallampati scores.
