# Supplementary material for: Is obstructive sleep apnea associated with difficult airway? Evidence from a systematic review and meta-analysis of prospective and retrospective cohort studies
Source: PLoS One. 2018 Oct 4;13(10):e0204904. doi: 10.1371/journal.pone.0204904 (PMC6171874; doi:10.1371/journal.pone.0204904)
Supplement: S5 File — (DOC) [file pone.0204904.s005.doc]

**Supplementary “S5 File”:** Study quality assessment

| **Study ID et al.** | **Study design** | **Study population clearly identified?** | **Clear definition of outcome and outcome assessment?** | **Selective loss of patients during the follow up?** | **Important confounders and / or prognostic factors identified** | **Newcastle-Ottawa scale scores** |
| --- | --- | --- | --- | --- | --- | --- |
| ^[12]^Hiremath^1998^ | RC | Yes | Yes | No | No | 7 |
| ^[20]^Brodsky^2002^ | PC | Yes | Yes | No | A larger neck circumference was associated with men (P <0.001), a higher Mallampati score (P = 0.0029), grade 3 views during laryngoscopy (P = 0.0375), and OSA (P = 0.0372) | 8 |
| ^[13]^Siyam^2002^ | RC | Yes | Yes | No | No | 7 |
| ^[21]^Sabers^2003^ | RC | Yes | No | No | No | 8 |
| ^[22]^Kheterpal^2006^ | PC | Yes | Yes | No | Multivariate regression analysis identified the following independent predictors of grade 3 or 4 MV combined with DI: limited or severely limited mandibular protrusion, thick/obese neck anatomy, history of sleep apnea, history of snoring, and BMI ≥30 kg/m^2^ | 8 |
| ^[15]^Kim^2006^ | RC | Yes | Yes | No | Age, height, weight, LSATPREOP, BMI, and hemoglobin were not significant confounding factors. | 9 |
| ^[23]^Chung^2008^ | PC | Yes | Yes | No | No | 6 |
| ^[24]^Kheterpal^2009^ | PC | Yes | Yes | No | The logistic regression model included 47,976 patients (91%) and demonstrated 5 independent predictors of impossible mask ventilation (p<0.05):  neck radiation changes, male sex, sleep apnea, Mallampati III or IV, and presence of beard. | 9 |
| ^[25]^Shah^2012^ | PC | Yes | No | No | The risk factors identified for DMV by univariate analysis were snoring, obstructive sleep apnea, retrognathia, micrognathia, macroglossia, edentulous teeth, short thick neck, Mallampatti grade [III/IV] abnormal SLUX grade, experience  level of anesthesiologist, Cormack Lehanne grade [III/IV], and BMI >26 kg/m^2^ | 8 |
| ^[14]^Ramachandran^2012^ | PC | Yes | Yes | No | Four independent predictors of uLMA™ failure were identified on logistic regression analysis: surgical table rotation (aOR 5.00), male sex (aOR 1.74), poor dentition (aOR 1.58), and increased body mass index (aOR 1.06 per unit body mass index increase) | 8 |
| ^[17]^Kheterpal^2013^ | PC | Yes | Yes | No | Age ≥46, male sex, body mass index ≥30 kg/m^2^, limited thyromental distance, Mallampati class III or IV, presence of beard, sleep apnea, presence of teeth, limited neck extension, limited jaw protrusion, thick neck, and neck radiation changes or neck mass all demonstrated statistical significance (P < 0.05) and clinical significance (adjusted odds ratio >1.5). The c-statistic of this non-parsimonious model was 0.84 (95% CI, 0.82–0.87), demonstrating good discriminating capacity. | 9 |
| ^[27]^Acar^2014^ | PC | Yes | Yes | No | No | 7 |
| ^[16]^Cattano^2014^ | RC | Yes | Yes | No | Seven independent risks factors for DMV were identified multivariate logistic regression model using stepwise selection: age ≥47 year, BMI ≥35 kg/m^2^, neck circumference ≥40 cm, history of difficult intubation, presence of facial hair, perceived short neck, and OSA; p<0.001. The model’s c-statistic is 0.75 (95% CI: 0.71-0.79), demonstrating a good discriminating capacity. | 9 |
| ^[19]^Corso^2014^ | PC | Yes | Yes | No | Adjusted risk factors for difficult intubation and difficult mask ventilation: age, gender, BMI etc. | 9 |
| ^[26]^Toshniwal^2014^ | PC | Yes | Yes | No | No | 8 |
| ^[18]^Gokay^2016^ | PC | Yes | Yes | No | No | 7 |

OSA = obstructive sleep apnea; PC = Prospective cohort; RC = Retrospective cohort; index.

**Supplementary “S5 File”: Study quality assessment by Newcastle-Ottawa scale**

| **Quality assessment**  **criteria** | **Acceptable** | ^[12]^Hire  math^1998^ | ^[20]^Brod  sky^2002^ | ^[13]^Si  yam^2002^ | ^[21]^Sa  bers^2003^ | ^[22]^Khe  terpal^2006^ | ^[15]^Kim^2006^ | ^[23]^Chung^2008^ | ^[24]^Khe  terpal^2009^ | ^[25]^Shah^2012^ | ^[14]^Rama  Chan  dran^2012^ | ^[17]^Kheterpal^2013^ | ^[27]^Acar^2014^ | ^[16]^Cattano^2014^ | ^[19]^Corso^2014^ | ^[26]^Toshniwal^2014^ | ^[18]^Gokay^2016^ |
| --- | --- | --- | --- | --- | --- | --- | --- | --- | --- | --- | --- | --- | --- | --- | --- | --- | --- |
| **Selection** | | | | | | | | | | | |  |  |  |  |  |  |
| **Representativeness**  **of exposed cohort?** | **Representative of average adult in**  **Preoperative period** | ***** | ***** | ***** | ***** | ***** | ***** | ***** | ***** | ***** | ***** | ***** | ***** | ***** | ***** | ***** | ***** |
| **Selection of the**  **non-exposed**  **cohort?** | **Drawn from same community as exposed**  **cohort** | ***** | ***** | ***** | ***** | ***** | ***** | ***** | ***** | ***** | ***** | ***** | ***** | ***** | ***** | ***** | ***** |
| **Ascertainment of**  **exposure?** | **Secured records, Structured interview, questionnaire** | ***** | ***** | ***** | ***** | ***** | ***** | ***** | ***** | ***** | ***** | ***** | ***** | ***** | ***** | ***** | ***** |
| **Demonstration that**  **outcome of interest**  **was not present at**  **start of study?** |  | ***** | ***** | **-** | ***** | ***** | ***** | **-** | ***** | ***** | ***** | ***** | ***** | ***** | ***** | ***** | ***** |
| **Comparability** | | | | | | | | | | | |  |  |  |  |  |  |
| **Study controls for**  **age/sex?** | **Yes** | **-** | **-** | ***** | ***** | **-** | ***** | **-** | ***** | **-** | **-** | ***** | **-** | ***** | ***** | ***** | **-** |
| **Study controls for**  **at least 3 additional**  **risk factors?** | **BMI, Neck circumference**  **etc** | **-** | ***** | **-** | **-** | ***** | ***** | **-** | ***** | ***** | ***** | ***** | **-** | ***** | ***** | **-** | **-** |
| **Outcome** | | | | | | | | | | | |  |  |  |  |  |  |
| **Assessment of**  **outcome?** | **Independent blind assessment, record linkage** | ***** | ***** | ***** | ***** | ***** | ***** | ***** | ***** | ***** | ***** | ***** | ***** | ***** | ***** | ***** | ***** |
| **Was follow-up long**  **enough for outcome**  **to occur?** | **Follow-up** | ***** | ***** | ***** | ***** | ***** | ***** | ***** | ***** | ***** | ***** | ***** | ***** | ***** | ***** | ***** | ***** |
| **Adequacy of**  **follow-up of**  **cohorts?** | **Complete follow-up, or subjects lost to**  **follow-up unlikely to introduce bias** | ***** | ***** | ***** | ***** | ***** | ***** | ***** | ***** | ***** | ***** | ***** | ***** | ***** | ***** | ***** | ***** |
| **Overall Quality Score**  **(Maximum = 9)** | | **7** | **8** | **7** | **8** | **8** | **9** | **6** | **9** | **8** | **8** | **9** | **7** | **9** | **9** | **8** | **7** |
