# Supplementary material for: Is obstructive sleep apnea associated with difficult airway? Evidence from a systematic review and meta-analysis of prospective and retrospective cohort studies
Source: PLoS One. 2018 Oct 4;13(10):e0204904. doi: 10.1371/journal.pone.0204904 (PMC6171874; doi:10.1371/journal.pone.0204904)
Supplement: S6 File — (DOC) [file pone.0204904.s006.doc]

**Supplementary “S6 File”: Definition of the airway outcomes in various study**

| **Study ID** | **Outcome definition** |
| --- | --- |
| ^[12]^Hiremath ^1998^ | Difficult intubation by an attending anesthesiologist with grade 4 Cormack and Lehane view |
| ^[20]^Brodsky ^2002^ | Tracheal intubation: classified as easy if the product of the graded laryngoscopy view times the number of intubation attempts was <3 and was classified as difficult when it was ≥3. |
| ^[13]^Siyam ^2002^ | Difficult intubation by the anesthesiologist with Grade 3 or 4 Cormack and Lehane view |
| ^[21]^Sabers ^2003^ | Data provided on the difficulty intubation. Outcome not defined |
| ^[22]^Kheterpal ^2006^ | Grade 3 (Difficult mask ventilation): defined as mask ventilation that is inadequate to maintain oxygenation, unstable mask ventilation, or mask ventilation requiring 2 providers.  Grade 4 mask ventilation: defined as impossible mask ventilation noted by absence of end-tidal CO_2_ measurement and lack of perceptible chest wall movement during positive pressure ventilation attempts despite airway adjuvants and additional personnel.  Direct laryngoscopy view: defined by Cormack and Lehane and >3 attempts by attending anesthesiologist and the inability to perform successful tracheal intubation using direct laryngoscopy. |
| ^[15]^Kim ^2006^ | Difficulty to intubate: intubation with grade 3 or 4 Cormack and Lehane view, when an intubation aid (stylet, intubating laryngeal mask airway, fiberoptic bronchoscope) was needed, or when 3 or more intubation attempts were required. |
| ^[23]^Chung ^2008^ | Difficult intubation: if on direct laryngoscopy grade 4 Cormack and Lehane view and required > 2 attempts to intubate. |
| ^[24]^Kheterpal ^2009^ | Impossible mask ventilation: defined as the inability to establish face-mask ventilation despite multiple airway adjuvants and two-hand mask ventilation  Difficult intubation: Grade 3 or 4 Cormack-Lehane direct laryngoscopy view or >3 attempts at intubation by an attending anesthesiologist. |
| ^[25]^Shah.^2012^ | Difficult mask ventilation: defined when signs of inadequate ventilation like gas leak around the mask, no perceptible chest movements, O_2_ saturation <92% by pulse oxymetry and alternative methods required to facilitate mask ventilation e.g. two-handed mask ventilation, insertion of oropharyngeal or nasopharyngeal airway and change of operator during general anesthesia.  Difficult intubation: Grade >2 Cormack Lehanne view on laryngoscopy. when the operator takes >2 attempts or 9 min for intubation. |
| ^[14]^Ramachandran ^2012^ | Laryngeal Mask Airway (LMA)™ failure, defined as any acute airway event occurring between insertion of LMA™ and completion of surgical procedure that required LMA™ removal and rescue endotracheal tube placement. |
| ^[17]^Kheterpal^2013^ | Difficult mask ventilation: Grade 3 or 4 mask ventilation. Direct Laryngoscope: Grade 3 or 4 Cormack-Lehane laryngoscopy view or ≥ 4 intubation attempts. Laryngoscopy view: inclusive of direct or video laryngoscopy. |
| ^[27]^Acar ^2014^ | Difficult intubation: Grade 3 or 4 Cormack and Lehane view, need for an intubation aid, or need for 3 or more intubation attempts. |
| ^[16]^Cattano^2014^ | Difficult mask ventilation: difficulty in maintaining a mask seal and obtaining a satisfactory capnography (end-tidal CO_2_ and tidal volume). If Ease of mask ventilation graded based on a severity score: from easy = 0, oral airway used = 1; to difficult, two handed ventilation = 2, or extraglottic device required = 3. |
| ^[19]^Corso.^2014^ | Difficult intubation was defined a) difficult laryngoscopy, defined as being characterized by the impossibility of obtaining a view of the vocal cords even after the best external laryngeal manipulation; b) necessity of repeated attempts; c) necessity of non-standard devices and/or procedures; d) withdrawal and procedure re-planning. |
| ^[26]^Toshniwal ^2014^ | Difficulty airway was classified 1) Difficult mask ventilation of Grade M3 (unable to maintain SpO2 >90%) during mask ventilation; 2) difficulty laryngoscopy of Grade B2 (more than one attempt and/or trauma to lips or buccal or pharyngeal mucosa); 3) Cormack and Lehane grade 3 or 4; and 4) difficulty intubation of Grade IF (one- time esophageal intubation or use of other devices to facilitate intubation, difficulty in endotracheal intubation). |
| ^[18]^Gokay ^2016^ | Cormack-Lehane classification (1-4) and difficult intubation grade Noted |
