# Supplementary figures and images for: Is obstructive sleep apnea associated with difficult airway? Evidence from a systematic review and meta-analysis of prospective and retrospective cohort studies
Source: PLoS One. 2018 Oct 4;13(10):e0204904. doi: 10.1371/journal.pone.0204904 (PMC6171874; doi:10.1371/journal.pone.0204904)

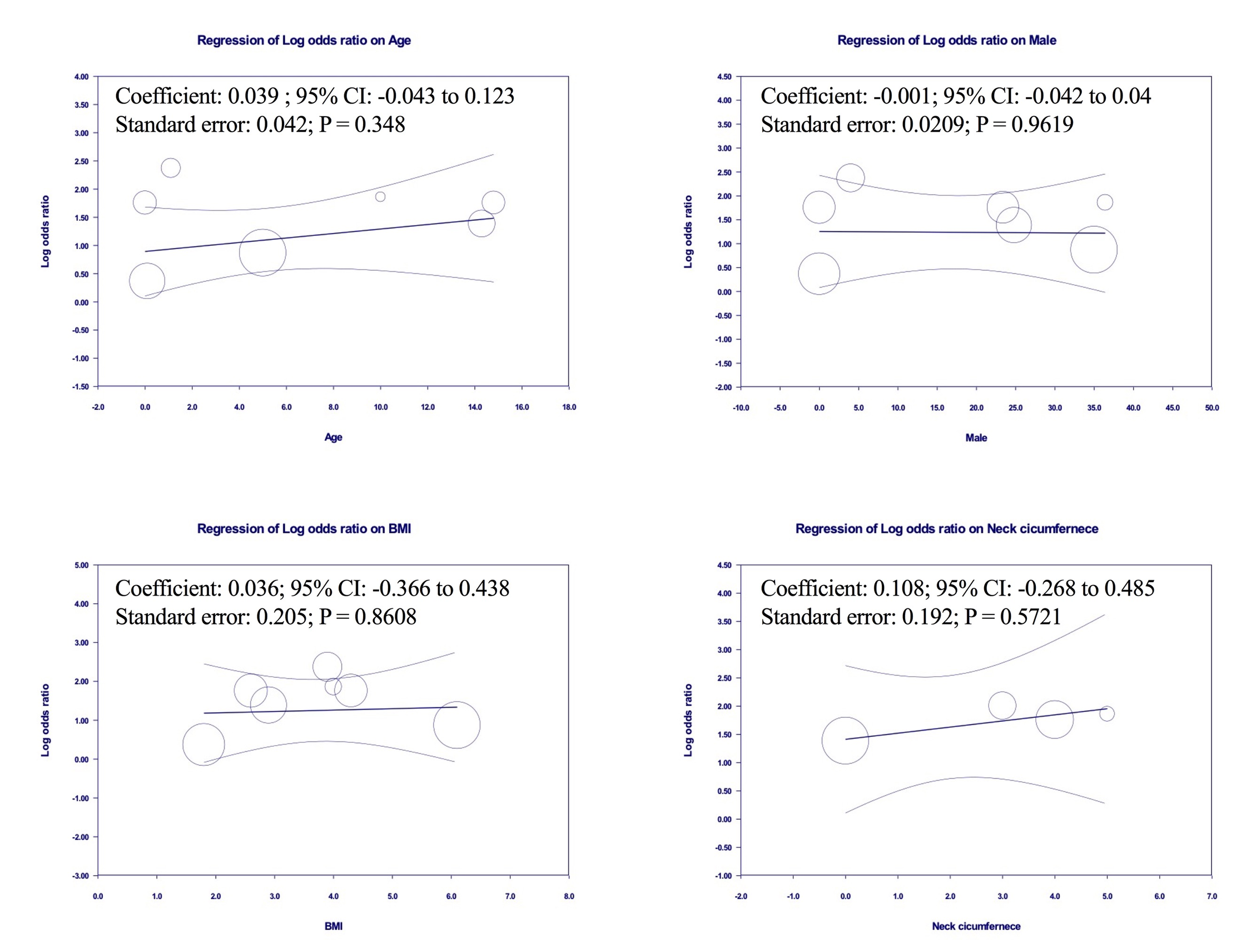

Supplement: S7 File — Each circle represents a study, telescoped by its weight in the analysis. The relationship was nonsignificant. (TIF) [file pone.0204904.s007.tif]
